# Incomplete thermal ablation-induced up-regulation of transcription factor nuclear receptor subfamily 2, group F, member 6 (NR2F6) contributes to the rapid progression of residual liver tumor in hepatoblastoma

**DOI:** 10.1080/21655979.2021.1945521

**Published:** 2021-07-24

**Authors:** Jin-shu Pang, Dong-yue Wen, Rong-quan He, Gang Chen, Peng Lin, Jin-hong Li, Yu-jia Zhao, Lin-Yong Wu, Jun-Hong Chen, Yun He, Li-Ting Qin, Jia-bo Chen, Yong Li, Hong Yang

**Affiliations:** a Department of Medical Ultrasonics, First Affiliated Hospital of Guangxi Medical University, Nanning, Guangxi Zhuang Autonomous Region, P.R. China; b Department of Medical Oncology, First Affiliated Hospital of Guangxi Medical University, Nanning, Guangxi Zhuang Autonomous Region, P.R. China; c Department of Pathology, First Affiliated Hospital of Guangxi Medical University, Nanning, Guangxi Zhuang Autonomous Region, P.R. China; d Department of Pathology, Maternal and Child Health Hospital of Guangxi Zhuang Autonomous Region, Nanning, Guangxi Zhuang Autonomous Region, P.R. China; e Department of Pediatric Surgery, First Affiliated Hospital of Guangxi Medical University, Nanning, Guangxi Zhuang Autonomous Region, P.R. China

**Keywords:** NR2F6, overexpression, hepatoblastoma, residual tumor, ablation

## Abstract

Hepatoblastoma is a kind of extreme malignancy frequently diagnosed in children. Although surgical resection is considered as the first-line treatment for hepatoblastoma, a relatively large population of patients have lost the preferred opportunity for surgery. Administration of locoregional ablation enables local tumor control but with the deficiency of insufficient ablation, residual tumor, and rapid progression. In this study, we integrated 219 hepatoblastoma and 121 non-cancer liver tissues to evaluate the expression of NR2F6, from which a higher NR2F6 level was found in hepatoblastoma compared with non-cancer livers with a standard mean difference (SMD) of 1.04 (95% CI: 0.79, 1.29). The overexpression of NR2F6 also appeared to be an efficient indicator in distinguishing hepatoblastoma tissues from non-cancer liver tissues from the indication of a summarized AUC of 0.90, with a pooled sensitivity of 0.76 and a pooled specificity of 0.89. Interestingly, nude mouse xenografts provided direct evidence that overexpressed NR2F6 was also detected in residual tumor compared to untreated hepatoblastoma. Chromatin immunoprecipitation-binding data in HepG2 cells and transcriptome analysis of HepG2 xenografts were combined to identify target genes regulated by NR2F6. We finally selected 150 novel target genes of NR2F6 in residual tumor of incomplete ablation, and these genes appeared to be associated with the biological regulation of lipid metabolism-related pathway. Accordingly, targeting NR2F6 holds a therapeutic promise in treating residual recurrent hepatoblastoma after incomplete ablation.

## GRAPHICAL ABSTRACT

**Figure:**
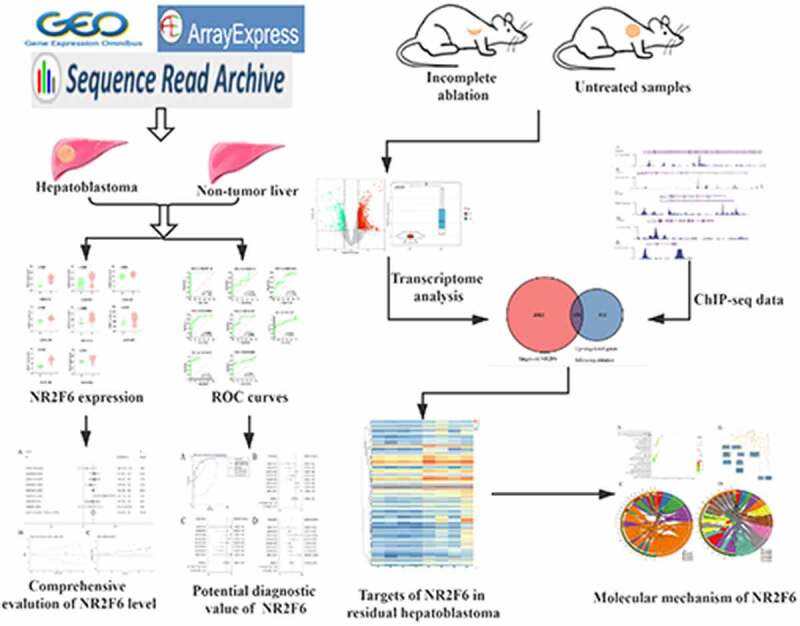

## 1 Introduction

Hepatoblastoma is the most prevalent hepatic tumor in children, accounting for 75% to 80% of pediatric liver malignancies [1]. Hepatoblastoma is a fetal-like tumor deriving from primordial embryonic cells believed to recapitulate liver growth and development [2,3]. In general, surgical resection is considered to be the preferred treatment for hepatoblastoma patients [4]. However, hepatoblastoma is characterized by rapid progression and invasiveness; more than half of the patients are diagnosed at an advancing stage, for which surgical resection delivers limited benefits, especially for patients with stage III/IV hepatoblastoma [5,6]. Conventional treatments, including surgery and combined chemotherapy, may not be suitable for the child suffering from metastatic and unresectable hepatoblastoma who has already received extensive therapies. For such patients, ultrasound-guided tumor ablation seems to be an alternative option. For such patients, ultrasound-guided tumor ablation seems to be an alternative option [7].

In recent years, ultrasound-guided tumor ablation has become the preferred clinical strategy for primary small liver tumors in adults because of the advantages of being minimally invasive with fewer complications compared to surgical resection [4,8]. But of note, some studies observed an important clinical phenomenon that recurrent residual tumor after incomplete ablation showed a more advanced ability on progression and invasiveness than recurrent tumor following surgery [9–12]. Increasingly successful cases with ultrasound-guided thermal ablation for recurrent or unresectable hepatoblastoma were reported during the past decade [7,11,13,14], while few studies currently focus on the clinical problem of residual hepatoblastoma after incomplete ablation, for which its underlying molecular mechanism remains largely unclear. Based on the emerging application of liver tumor ablation, in-depth research to reveal the mechanism of proliferation and invasion of residual cancer cells after incomplete ablation is about to promote precise medical treatment for patients with residual hepatoblastoma.

Transcription factor nuclear receptor subfamily 2, group F, member 6 (NR2F6) is a member of a nuclear receptor superfamily that has been shown to be crucial for the regulation of biological events, such as metabolism, reproduction, and development [15]. Several reports have underscored the emphasis of NR2F6 in human cancers [16–20]. For head and neck squamous cell carcinoma (HNSCC), a higher NR2F6 protein level had been detected in primary tumors that developed into locally recurrent tumors compared with nonrecurrent primary cancers, indicating high expression of NR2F6 protein may be a useful biomarker for early prediction of local recurrences in HNSCC [19]. Besides, a study published in 2018 provided direct evidence to manifest that NR2F6 functioned as an intracellular immunity checkpoint, and genetic elimination of NR2F6 enables improved PD-L1 blockade activity of immunotherapy [20]. Nevertheless, whether NR2F6 is a direct factor to incite more progression of residual hepatoblastoma justifies further investigation.

In this study, we confirmed a higher expression of NR2F6 in 219 cases of hepatoblastoma than 121 cases of non-cancer child liver by combining high throughput RNA sequencing (RNA-seq) data. We therefore hypothesized that overexpressed NR2F6 might contribute to the carcinogenesis of hepatoblastoma. The aim of this paper is to investigate the role of NR2F6 in the progression of residual tumor. The nude mouse xenografts were performed, which indicated an upregulated NR2F6 level in the ablation xenografts after incomplete ablation. Furthermore, targets regulated by the transcription factor NR2F6 were identified by integrating ChIP-seq and nude mouse xenograft models. These findings supported that NR2F6 might be a prospective therapeutic target for recurrent residual hepatoblastoma after insufficient ablation.

## 2. Materials and methods

### 2.1 Evidence-based samples for evaluation of the NR2F6 level

To systematically evaluate the expression level of NR2F6 in hepatoblastoma, we tried to obtain available samples from several public databases (GEO, SRA, and ArrayExpress) through the searching words of hepatoblastoma. The samples that met the following criteria would be included [1]: NR2F6 expression was detected by well-established methods, such as PCR, microarrays, RNA-seq, et al. [2]; samples derived from human hepatoblastoma and liver tissues rather than xenografts [3]; tissues for both hepatoblastoma and liver tissues should not be less than three samples [21].

### 2.2 Nude mouse xenograft model

In order to explore the underlying mechanisms of NR2F6 in residual hepatoblastoma, nude mouse xenograft experiments were performed to simulate incomplete ablation of human hepatoblastoma. Human hepatoblastoma (HepG2) cell line was provided by Shanghai Institutes for Biological Sciences, Chinese Academy of Sciences. HepG2 cells were cultured in 25 cm^2^ dishes containing Dulbecco’s Modified Eagle’s Medium (Thermo Scientific, Inc.) supplemented with 10% fetal bovine serum and 1% streptomycin-penicillin antibiotic mixture in an atmosphere of 5% CO_2_ at 37°C for 7 days. And specific pathogen-free BALB/c nude mice (N = 8; female; 6 weeks; 8–22 g) were purchased from Shanghai SLAC Laboratory Animal Co., Ltd. *Of note, the ove* [22]. Nude mice were randomly separated into two groups when the transplanted tumor grew to around 10 mm. After nude mice were anesthetized with 1% pentobarbital, the experimental group (N = 4) underwent treatment of incomplete ablation through the Cool-tip™ radiofrequency ablation system (power: 30 W; time: 10 s; temperature: 70 ± 5°C). Meanwhile, controls (N = 4) were treated by the Cool-tip™ radiofrequency ablation system (power: 0 W; time: 10 s; temperature: 20 ± 5°C). Theoretically, genetic changes occur in the cancer cells around the ablation site when cells are subjected to thermal stimulation, and the cancer cells of incomplete ablation show more aggressive progression than untreated cells. The previous publications have proven that this experiment can imitate the biological properties similar to residual liver tumor observed clinically [22–25]. Following another 24-h culture, HepG2 xenografts were removed and immediately protected by RNAsafer Stabilizer Reagent (OMEGA, Guangzhou, China) for RNA-seq detection. Differentially expressed genes between the incomplete ablation group and untreated controls were analyzed using the limma package of R language. Differently expressed genes (DEGs) would be selected with a statistic p-value < 0.05 and a log2|fold-change (FC)|>1. Additionally, this experiment was approved by the Ethics Committee of the First Affiliated Hospital of Guangxi Medical University (Nanning, China).

### 2.4 Statistical analysis

Statistical analysis was performed using Prismpad 8.0 and R language. All raw expression data were normalized into a log _2_(x + 1) scale, and gene expression levels were calculated into mean ± SD. Students’ t-tests were used to examine expression differences between the two groups. And for systematic evaluation of the NR2F6 level in hepatoblastoma, evidence-based evaluation models, including a fixed-effects model and a random-effects model, were constructed using the Stata 12.0 software. When heterogeneity >50%, a random-effects model would be used; otherwise, a fixed-effects model would be the preferred option. Then, publication bias existing in evidence-based models was further assessed using Begg’s and Egger’s tests. Further, latent competence for NR2F6 to differentiate between hepatoblastoma and non-tumor liver tissues was also assessed by pooled sensitivity and specificity, together with a summarized receiver operating characteristic (ROC) curve [26,27].

### 2.5 Targets of NR2F6 in hepatoblastoma HepG2 cells

Transcription factors usually exert a regulatory influence on gene expression by binding to promoter regions. Chromatin immunoprecipitation (ChIP) assay is a helpful technique to explore interactions of protein-DNA and to advance epigenetic modifications and genetic regulation. ChIP-seq technique can identify and relatively measure-specific interactions between protein and chromatin at multiple loci in the human genome. Based on CistromeDB, four ChIP-seq datasets of NR2F6 (CistromeDB: 63,772, 63,771, 101,379, and 101,378) were obtained to screen latent targets regulated by NR2F6 in the hepatoblastoma HepG2 cells. The genes with interaction scores greater than 2 and repeated in at least two datasets among four ChIP-seq analyses were considered as potential targets for NR2F6 [28].

### 2.6 Underlying mechanism of NR2F6 in residual hepatoblastoma

In this study, transcriptome analysis of incomplete ablation xenograft and NR2F6 ChIP-seq data were jointly used to detect the latent genes targeted by NR2F6 in residual hepatoblastoma. We selected the intersection genes appearing at upregulated DEGs following incomplete ablation and the targets of NR2F6 detected by ChIP-seq. To reveal the underlying mechanism of NR2F6 in recurrent residual hepatoblastoma, Gene Ontology (GO) annotation and pathway analysis including Reactome pathway and Kyoto Encyclopedia of Genes and Genomes (KEGG) pathway were finally carried out using WebGestalt server [29].

In this work, increased NR2F6 was determined from hepatoblastoma based on large-scale data including 440 samples. Subsequently, hepatoblastoma-derived HepG2 cells were inoculated under the right armpit of mice. Treatment of incomplete ablation was performed for HepG2 xenografts, from which a higher NR2F6 level was detected in residual hepatoblastoma untreated samples. A total of 150 targets regulated by NR2F6 in residual tumor were identified from experimentally verified ChIP-seq data and transcriptome analysis, and a molecular mechanism for these genes was disclosed utilizing GO annotation and pathway analysis, indicating NR2F6 along with its targets greatly contribute to the dysregulation of biological activity related to lipid metabolism.

## 3 Results

### 3.1 Overexpression of NR2F6 in hepatoblastoma

**Figure 1.**
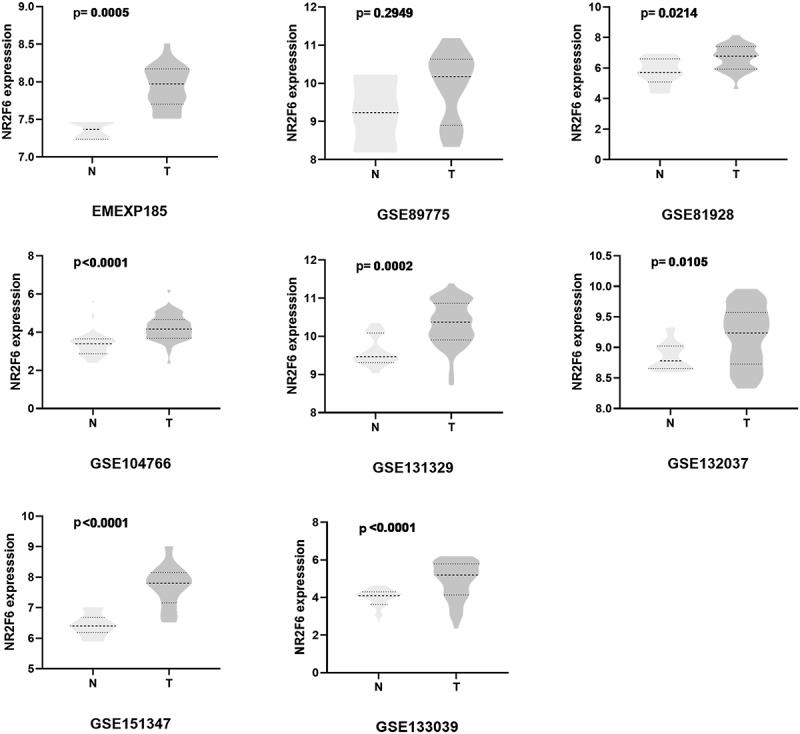
NR2F6 expression between hepatoblastoma and normal livers in per studies

**Figure 2.**
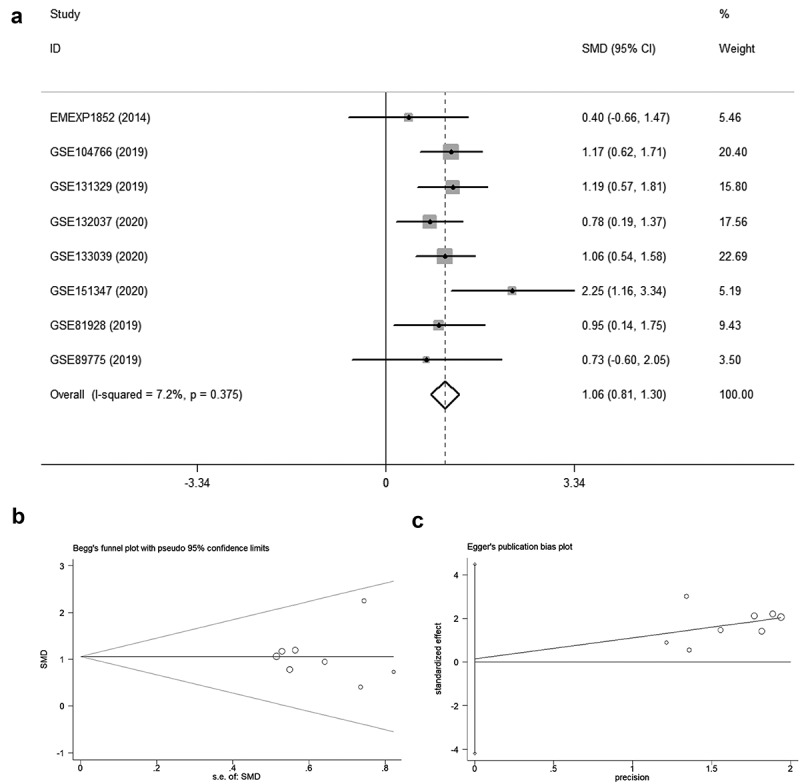
The overall evaluation of NR2F6 expression. (a) The pooled level of NR2F6 expression between hepatoblastoma and normal livers with 440 samples and detection of publication bias of Begg’s (b) and Egger’s plots (c)

In seven datasets among nine studies, NR2F6 exhibited clearly higher levels in hepatoblastoma tissues than non-cancer child livers (p < 0.05; Table 1, Figure 1). Further, an evidence-based model, including 219 hepatoblastoma samples and 121 non-cancer liver tissues, was performed to assess the expression of NR2F6, from which NR2F6 was also found to be overexpressed in hepatoblastoma compared with non-cancer liver with a standard mean difference (SMD) of 1.04 (95% CI: 0.79, 1.29; heterogeneity: 1%; random-effects model; Figure 2(a)). Additionally, there is no significant publication bias detected from Begg’s and Egger’s tests, which further strengthens the credibility of this model for the measurement of NR2F6 expression (p > 0.05; Figure 2(b,c)).

**Table 1.** Included studies for evaluation of differentially expressed genes in hepatoblastoma

Datasets	Update Year	Country	Methods	Ca. N	Ca. Mean	Ca. SD	Ctal. N	Ctal. Mean	Ctal. SD
E-MEXP-1852	2014	France	Microarray	25	7.69	0.4617	4	7.51	0.2781
GSE104766	2019	France	RNA-seq	30	4.23	0.7728	30	3.38	0.6832
GSE131329	2019	Japan	Microarray	53	10.3	0.6151	14	9.61	0.4076
GSE132037	2020	Spain	Microarray	34	9.19	0.4928	18	8.86	0.2342
GSE133039	2020	Spain	RNA-seq	33	4.87	1.0734	32	3.98	0.4945
GSE151347	2020	Germany	RNA-seq	11	7.73	0.7281	11	6.43	0.3717
GSE81928	2019	USA	RNA-seq	23	6.63	0.8835	9	5.79	0.8992
GSE89775	2019	USA	RNA-seq	10	9.93	0.9598	3	9.22	1.052

### 3.2 Potential clinical effectiveness of NR2F6 in hepatoblastoma

**Figure 3.**
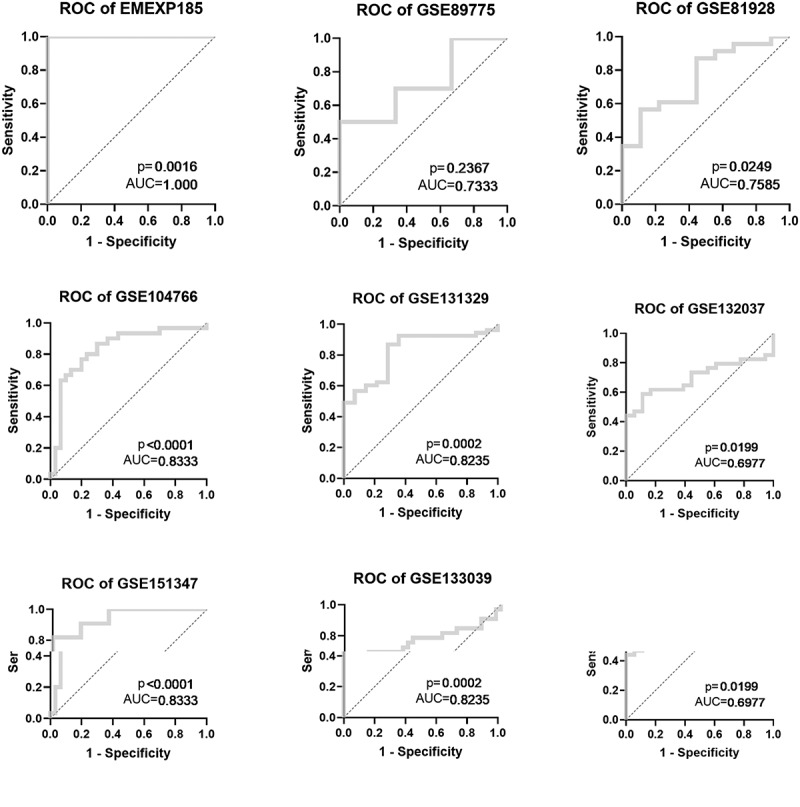
ROC curves indicating the latent value of NR2F6 in the diagnosis of hepatoblastoma in per studies

**Figure 4.**
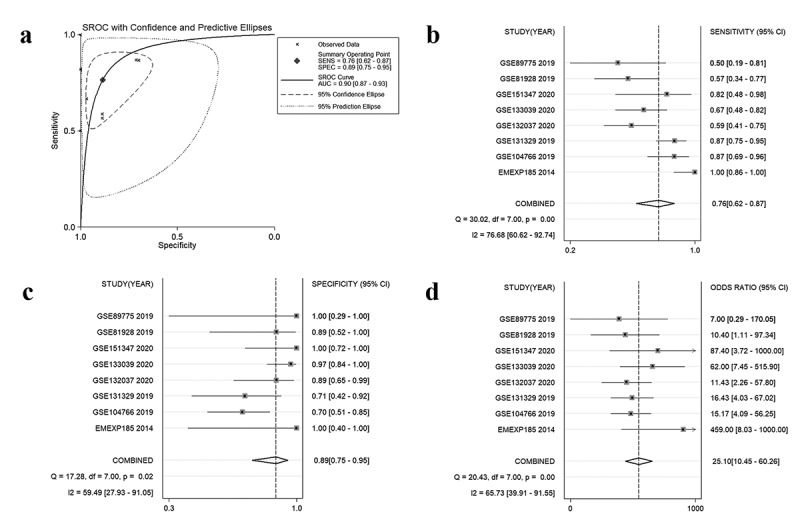
The overall evaluation of latently diagnostic value of NR2F6 in hepatoblastoma. (a) Summarized ROC curve with 440 samples and pooled forest plots of sensitivity (b) and specificity (c) in distinguishing hepatoblastoma and normal livers, as well as the odds ratio of NR2F6 between hepatoblastoma and normal livers

After the evaluation of NR2F6 expression, the potential diagnostic effectiveness of increased NR2F6 level in hepatoblastoma was further explored with nine included studies. Based on the ROC curves for each dataset, the results (AUC> 0.7) of eight studies indicated that NR2F6 had a relatively high ability to differentiate between hepatoblastoma and non-cancer child livers (Figure 3). Besides, the overall AUC of 0.9 (Figure 4(a)) further revealed that overexpressed NR2F6 might act as a potential biomarker to distinguish hepatoblastoma and non-cancer liver tissues with a sensitivity of 0.76 (Figure 4(b)) and a specificity of 0.89 (Figure 4(c)), suggested by the summarized ROC integrated by 440 samples. Odds ratio (OR) forest plot also suggested that the population with upregulated NR2F6 levels was more likely to suffer from hepatoblastoma (OR = 25.1; 95% CI:10.45, 60.26; Figure 4(d)).

### 3.3 Targets of NR2F6 in residual tumor

**Figure 5.**
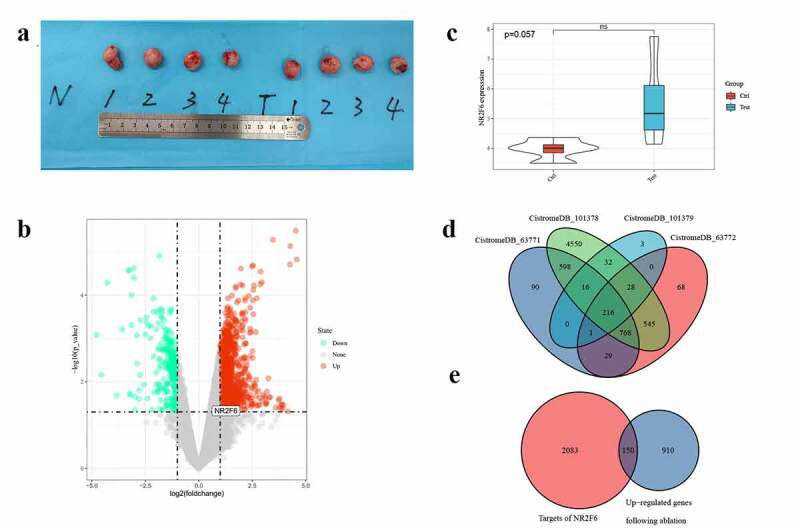
Determination of potential targets in residual hepatoblastoma after incomplete ablation. (a) ablation-treated model group (T, N = 4) and normal untreated group (N, N = 4); (b) differentially expressed analysis of ablation-treated model and untreated group; (c) an upregulated tendency of NR2F6 expression induced by incomplete ablation; (d) selection of potential targets using 4 datasets of ChIP-seq detection in liver cancer HepG2 cells; and (e) the latent targets regulated by NR2F6 in residual hepatoblastoma treated by incomplete ablation

**Figure 6.**
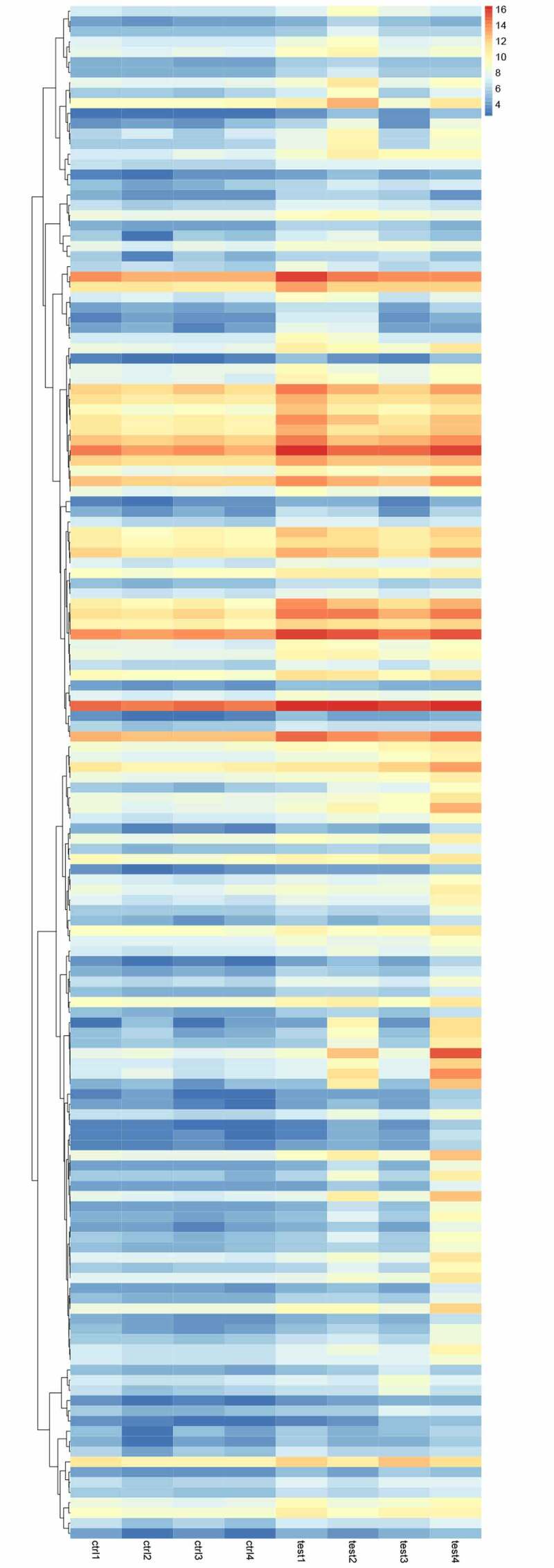
The heatmap displaying the level of 150 potential targets regulated by NR2F6 in residual hepatoblastoma after incomplete ablation. Note: the ‘test’ represents the ablation-treated model group, and ‘Ctal’ indicates the untreated group

Compared with untreated samples, 1,060 genes were upregulated and 289 genes underexpressed in residual hepatoblastoma after incomplete ablation (Figure 5(a-b)). Of note, the overexpression of NR2F6 was also detected in residual tumor (log_2_FC = 1.590, p = 0.03), suggesting that NR2F6 might also play an important role in residual tumor. Besides, an upward trend of NR2F6 expression could still be observed in residual tumor when compared to an untreated group, although nonparametric t tests did not indicate a significantly statistical p-value (p = 0.057; Figure 5(c)). Meanwhile, NR2F6 protein was found to interact with 2,233 target genes in hepatoblastoma HepG2 cells from 4 ChIP-seq studies (Figure 5(d)). To reveal the role of NR2F6 in residual tumor, we selected the overlaps that were both upregulated in residual tumor and targeted by NR2F6, and we eventually figured out 150 promising genes regulated by NR2F6 in residual tumor (Figure 5(e)). The expression of these genes in HepG2 xenografts is displayed in Figure 6.

### 3.4 Molecular mechanism of genes targeted by NR2F6

**Figure 7.**
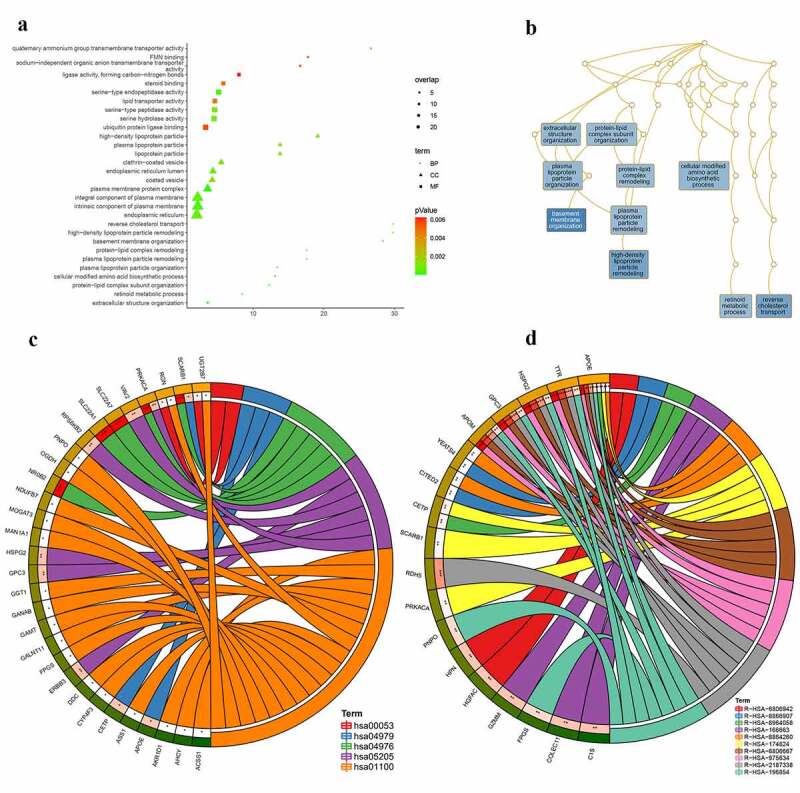
Molecular mechanism for the 150 potential targets regulated by NR2F6 in residual hepatoblastoma after incomplete ablation. (a) Gene ontology analysis, including biological process, cellular component, and molecular function, (b) interactions of biological processes, as well as KEGG (c) and reactome (d) pathway analysis

**Figure 8.**
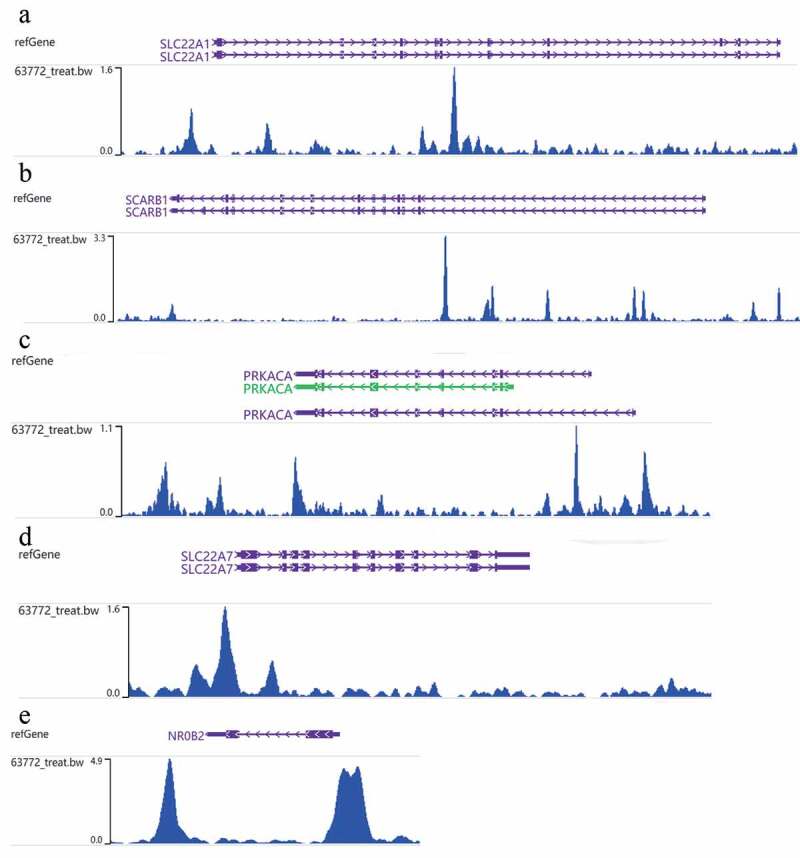
Validation for the targets of NR2F6 involved in KEGG pathway of bile secretion

According to GO analysis (**Table S1;** Figure 7(a)), the genes targeted by NR2F6 in residual hepatoblastoma were clearly involved in the processes of basement membrane organization, reverse cholesterol transport, and high-density lipoprotein particle remodeling (biological process; Figure 7(b)). Besides, for a cellular component, proteins encoded by these genes seem to be the structure of the plasma membrane protein complex, an intrinsic component of the plasma membrane, and an integral component of the plasma membrane. From molecular function annotation, these genes were shown to participate in the activity of serine-type endopeptidase, serine-type peptidase, serine hydrolase, et al. Further, as suggested by both KEGG and Reactome pathways (Table 2; Figure 7(c,d)), the upregulated NR2F6 and its targets notably exert a crucial influence on metabolism-related pathways for residual hepatoblastoma, especially cholesterol metabolism, retinoid metabolism and transport, and lipid-soluble vitamins metabolism. In addition, the ChIP-seq peak of NR2F6 for the targets involved in the KEGG bile secretion pathway is displayed in Figure 8.

**Table 2.** Pathway analysis for the 150 potential targets regulated by NR2F6 in ablation-treated residual tumor of hepatoblastoma

Geneset	Terms	Overlap	P-value	Symbol
hsa04976	Bile secretion	5	0.000	NR0B2;SCARB1;SLC22A1;SLC22A7; PRKACA
hsa05205	Proteoglycans in cancer	6	0.009	VAV2;ERBB3;RPS6KB2;GPC3;HSPG2; PRKACA
hsa04979	Cholesterol metabolism	3	0.010	APOE;SCARB1;CETP
hsa00053	Ascorbate and adorate metabolism	2	0.025	RGN;UGT2B7
hsa01100	Metabolic pathways	18	0.039	AHCY;GGT1;AKR1D1;GALNT11; GANAB;NDUFB7;RGN;ACSS1;ASS1; CYP4F3;DDC;FPGS;GAMT;MAN1A1; MOGAT3;OGDH;PNPO;UGT2B7
R-HSA-975,634	Retinoid metabolism and transport	5	0.000	APOE;TTR;APOM;GPC3;HSPG2
R-HSA-6,806,667	Metabolism of fat-soluble vitamins	5	0.000	APOE;TTR;APOM;GPC3;HSPG2
R-HSA-2,187,338	Visual phototransduction	6	0.000	APOE;TTR;APOM;GPC3;HSPG2;RDH5
R-HSA-166,663	Initial triggering of complement	3	0.001	COLEC11;GZMM;C1S
R-HSA-6,806,942	MET Receptor Activation	2	0.001	HPN;HGFAC
R-HSA-196,854	Metabolism of vitamins and cofactors	7	0.002	APOE;TTR;APOM;FPGS;GPC3;HSPG2; PNPO
R-HSA-8,964,058	HDL remodeling	2	0.004	APOE;CETP
R-HSA-174,824	Plasma lipoprotein assembly, remodeling, and clearance	4	0.004	APOE;SCARB1;CETP;PRKACA
R-HSA-8,866,907	Activation of the TFAP2 (AP-2) family of transcription factors	2	0.006	CITED2;YEATS4
R-HSA-8,864,260	Transcriptional regulation by the AP-2 (TFAP2) family of transcription factors	3	0.006	APOE;CITED2;YEATS4

## 4 Discussion

Ultrasound-guided thermal ablation has become a first-line option that has made great progress in treating liver tumors because of minimally invasive injury and a lower rate of complications than surgical resection [4,8,30,31]. In recent years, many kinds of research have manifested the potent advantages of minimally invasive ablation for liver tumors [32–34]. However, of note, ultrasound-guided thermal ablation enables locoregional treatment for hepatic tumors, but with the weaknesses of recurrent residual tumors and inferior outcomes caused by incomplete ablation [35–37]. Although several studies have aimed to elucidate the mechanism of worse malignancy for recurrent residual liver tumor, more specific molecular indicators still await exploration [38–40]. In the current study, overexpressed NR2F6 in hepatoblastoma tissues was confirmed through 440 samples. Subsequently, a higher NR2F6 was also detected in residual tumor following insufficient ablation. We therefore deduced the important role of NR2F6 in residual liver tumor of incomplete ablation, and further transcriptome analysis and ChIP-seq data were combined to probe its potential molecular mechanism.

Gene transcription of eukaryotes relies on the regulation of transcription factors. Nuclear receptors, a superfamily of transcription factors, are distributed in a variety of human tissues and organs and are widely involved in many kinds of physiological events, such as cell growth, development, and metabolism [41–43]. The characterization of nuclear receptors has a strong history in drug discoveries and encourages the development of precise therapy targeting these receptors for several diseases, including cancer, autoimmunity, and atherosclerosis [44–46]. NR2F6, a member of nuclear receptors, has been suggested to play a crucial role in several human cancers [16–20]. Liu’s study indicated that NR2F6 overexpression was related to poor overall survival and also stimulated DDA1 transcription by binding to the promoter region in ovarian cancer [47]. For hepatocellular carcinoma (HCC), the transcription of NR2F6 could be activated by circRHOT1 and therefore promote the progression of patients with HCC [48]. However, the biological role of NR2F6 residual liver tumor after incomplete ablation has not been reported.

In the current research, a higher NR2F6 expression was confirmed in hepatoblastoma than non-cancer liver with a combination of RNA-seq and microarrays samples, including 219 hepatoblastoma tissues and 121 non-cancer liver tissues (SMD = 1.04; 95% CI: 0.79, 1.29). At the same time, the overexpression of NR2F6 appeared to be an efficient indicator in distinguishing hepatoblastoma tissues from non-cancer liver tissues from the indication of summarized AUC of 0.90, with a pooled sensitivity of 0.76 and a pooled specificity of 0.89, demonstrating that overexpressed NR2F6 might act as a promising biomarker in the diagnosis of hepatoblastoma. Nevertheless, whether serum NR2F6 could be used to diagnose NR2F6 in clinical practice needs an in-depth investigation. To further study the role of residual liver tumor, hepatoblastoma HepG2 cells were subcutaneously transplanted into nude mice. Interestingly, increased NR2F6 was also found in the group treated by incomplete ablation in comparison with the untreated group. This finding provides a shred of novel evidence to reveal the underlying mechanism of residual hepatoblastoma.

To elucidate how NR2F6 functions as a transcription factor in residual hepatoblastoma, differentially expressed analyses were performed between incomplete ablation xenografts and an untreated group, from which we selected 1,060 upregulated genes through the threshold of a p-value < 0.05 and a log2FC > 1. Subsequently, genome-wide binding data (ChIP-seq) in HepG2 cells were combined to identify which genes could be bound by NR2F6. Finally, NR2F6 was found to clearly interact with the promoter region of 150 targets among 1,060 upregulated genes after incomplete ablation. According to the GO annotation and pathway analysis, these 150 genes targeted by NR2F6 in residual tumor are apparently related to the biological events of a metabolism-related pathway, especially lipid metabolism.

Lipid metabolic reprogramming is an essential property of tumor cells and has recently drawn much attention from researchers. A growing number of studies have shown that nuclear receptors play a key role in the regulation of lipid metabolism reprogramming, such as NR4A1, which serves as a key transcriptional regulator of liposome and glucose homeostasis, as well as lipid metabolism [49]. Lipid metabolism acts as signal regulatory molecules, greatly affecting cell function on the proliferation, apoptosis, and differentiation of normal or tumor cells [50]. It is well established that the biological behaviors of tumor cells, including liver tumor cells, are closely related to enhanced lipid metabolisms, such as lipid uptake, which contributes to the rapid growth of tumor cells and tumor development [50]. The activation of lipid synthesis, considered to be a sign of tumor cell invasiveness, is required for rapid proliferation of tumor cells and is also related to alteration of intracellular oncogenic signals and endoplasmic reticulum homeostasis [50–52]. Moreover, tumor cells with higher lipid content are easier to adapt to harmful stimuli (such as free radicals and chemotherapy) and thus reduce apoptosis induced by these stimuli [53,54]. Contrarily, inhibition of lipid synthesis helps to reduce tumor proliferation and invasion and enables cell death exposed by oxidative stress [53,54]. Therefore, the dysfunction of lipid metabolism influenced by NR2F6 might be a key factor that contributes to the increased malignancy of residual liver tumor after incomplete ablation.

Despite the aforementioned findings, some deficiencies should still be illustrated. First, the expression evaluation of NR2F6 is based only on microarrays and high throughput sequencing. An additional detection technique, such as qRT-PCR, needs to be performed. Besides, although increased levels of NR2F6 mRNA were detected in both hepatoblastoma and residual tumor, changes of NR2F6 protein should be further validated by Western blot or immunochemistry. In addition, the targets of NR2F6 in residual hepatoblastoma were significantly related to the regulation of lipid metabolism as indicated by GO annotation and pathway analysis. Therefore, we mainly focused on and mainly discussed the lipid metabolic regulation of NR2F6 in residual hepatoblastoma. However, whether these targets regulated by NR2F6 would be involved in cell-cycle progression, cell apoptosis, and angiogenesis needs to be experimentally verified by evidence in our further research.

## 5. Conclusion

We are the first to confirm the overexpression of NR2F6 in hepatoblastoma tissues, utilizing large-sample evaluation from multi-region and multi-source samples. Besides, we are also the first to find the increased level in residual tumor received incomplete ablation compared with untreated xenografts. Through ChIP-seq data and transcriptome analysis, the targets and molecular mechanism for NR2F6 were disclosed by GO annotation and pathway analysis, indicating that the rapid progression of residual hepatoblastoma might result from dysregulation of lipid metabolism stimulated by overexpressed NR2F6 in residual hepatoblastoma. In the future, an in-depth investigation will be performed to validate our findings.

## Supplementary Material

Supplemental Material

## Data Availability

Data supporting the results were obtained from several public databases (GEO, SRA, and ArrayExpress) and in-house nude mouse xenograft experiment. E-MEXP-1852(https://www.ebi.ac.uk/arrayexpress/experiments/E-MEXP-1852/); GSE104766(https://www.ncbi.nlm.nih.gov/geo/query/acc.cgi); GSE131329(https://www.ncbi.nlm.nih.gov/geo/query/acc.cgi); GSE132037(https://www.ncbi.nlm.nih.gov/geo/query/acc.cgi); GSE133039(https://www.ncbi.nlm.nih.gov/geo/query/acc.cgi); GSE151347(https://www.ncbi.nlm.nih.gov/geo/query/acc.cgi) GSE81928(https://www.ncbi.nlm.nih.gov/geo/query/acc.cgi); GSE89775(https://www.ncbi.nlm.nih.gov/geo/query/acc.cgi)

## Highlight:

1. This is the first study to indicate the upregulated expression of NR2F6 in hepatoblastoma with multiregion and multisource samples.
2. A higher level of NR2F6 was found in residual hepatoblastoma xenografts receiving incomplete ablation.
3. Upregulated NR2F6 contributes to the dysregulation of lipid metabolism in residual hepatoblastoma.
